# Evaluation of Perineal Descent Measurements on Pelvic Floor Imaging

**DOI:** 10.3390/jcm14020548

**Published:** 2025-01-16

**Authors:** Isabelle M. A. van Gruting, Kirsten Kluivers, Aleksandra Stankiewicz, Joanna IntHout, Kim W. M. van Delft, Ranee Thakar, Abdul H. Sultan

**Affiliations:** 1 Department of Obstetrics and Gynaecology, Croydon University Hospital, Croydon CR7 7YE, UK; ranee.thakar@nhs.net (R.T.);; 2 Department of Obstetrics and Gynaecology, Radboud University Medical Center, 6525 GA Nijmegen, The Netherlands; 3 Department of Radiology, Croydon University Hospital, Croydon CR7 7YE, UK; ola.stankiewicz@uhnm.nhs.uk; 4 Department of Radiology, University Hospitals of North Midlands NHS Trust, Stoke-on-Trent ST4 6QG, UK; 5 IQ Health Department, Radboud University Medical Center, 6525 GA Nijmegen, The Netherlands; 6 Department of Obstetrics and Gynaecology, Jeroen Bosch Ziekenhuis, 5223 GW Den Bosch, The Netherlands; 7 Medical School, St. Georges University London, London SW17 0RE, UK

**Keywords:** perineal descent, obstructed defaecation syndrome, diagnostic test accuracy, pelvic floor ultrasound

## Abstract

**Objectives:** The aim of this study is to validate a uniform method for measuring perineal descent which can be used for different imaging methods, to establish cut-off values for this measurement, and to assess diagnostic test accuracy (DTA) of imaging techniques using these cut-off values. Secondly, the study aims to correlate perineal descent to symptoms, signs and imaging findings in women with obstructed defaecation syndrome (ODS) to assess its clinical relevance. **Methods:** Cross-sectional study of 131 women with symptoms of ODS. Symptoms and signs were assessed using validated methods. These women underwent evacuation proctography (EP), magnetic resonance imaging (MRI), transperineal ultrasound (TPUS) and endovaginal ultrasound (EVUS). Perineal descent was measured on EP and MRI as the position of anorectal junction (ARJ) with respect to the pubococcygeal line (PCL) at rest (i.e., static descent) and during evacuation (i.e., descent at Valsalva). Dynamic perineal descent was measured on all four imaging techniques as the difference between the position of the ARJ at rest and Valsalva. DTA of dynamic perineal descent was estimated using Latent Class Analysis in the absence of a reference standard. **Results:** Interobserver agreement of dynamic perineal descent measurements was good for MRI and EVUS (ICC 0.86 and 0.85) and moderate for EP and TPUS (ICC 0.61 and 0.59). The systematic differences in measurements between imaging techniques show the need for individual cut-off values. New established cut-off values for dynamic descent are for EP 20 mm, MRI 35 mm, TPUS 15 mm and EVUS 15 mm. Sensitivity was moderate for EP (0.78) and MRI (0.74), fair for TPUS (0.65) and poor for EVUS (0.58). Specificity was similar for all imaging techniques (0.73–0.77). Static perineal descent correlated with symptoms of pelvic organ prolapse (POP) (*r* = 0.19), prolapse of all three compartments (*r* = 0.19–0.36), presence of levator ani muscle avulsion (*p* = 0.01) and increased hiatal area (*r* = 0.51). Dynamic perineal descent correlated with excessive straining (*r* = 0.24) and use of laxatives (*r* = 0.24). Classic symptoms of ODS (incomplete evacuation and digitation) did not correlate with perineal descent measurements. Static and dynamic perineal descent were associated with presence of rectocele, enterocele, intussusception, and absence of anismus. **Conclusions:** Dynamic perineal descent is a reliable measurement that can be applied to different imaging techniques to allow standardisation. Static descent is more often present in women with POP and dynamic descent is more often present in women with constipation. Perineal descent does not correlate with typical symptoms of ODS. Specificity of TPUS and EVUS is comparable to EP and MRI, hence ultrasound could be used for the initial assessment of pelvic floor dysfunction.

## 1. Introduction

Obstructed defaecation syndrome (ODS) is described as difficulty in evacuation of stools from the rectum, causing the feeling of incomplete evacuation, excessive straining and the need to manually assist defaecation [1]. In contrast to functional constipation, it can be caused by physical blockage of the faecal stream due to anatomical disorders. The posterior pelvic floor disorders rectocele, enterocele, intussusception and anismus have been clearly identified as causes of ODS, however little is known about the role of perineal descent.

The descending perineum syndrome is defined on clinical examination as the abnormal descent or ballooning (beyond the ischial tuberosities) of the perineum during straining (i.e., perineal descent) [2]. Possible causes of this condition are thought to be excessive and repetitive straining, and weakness of the pelvic floor muscles caused by either neuropathic degeneration of the muscles due to age, or trauma to the muscles or nerve supply during pregnancy and childbirth [2,3,4]. Currently, there is no surgical treatment, however timely identification is important as excessive perineal descent could cause stretch injury (denervation) to the pudendal nerves and sacral roots leading to neuropathic faecal incontinence [4,5].

There are different methods to locate the perineal position on imaging; e.g., the puborectalis muscle [6,7,8], the caudal margin of the anal sphincter [9,10,11] and the anorectal junction (ARJ) have been described [3,4,6,12,13,14,15]. A recent consensus of experts agreed the ARJ should be used, as this landmark is most consistent both at rest and during evacuation [16]. A lack of consensus exists regarding which anatomical structure can be used as the reference point to measure perineal descent; e.g., the level of the ischial ramus [17], tuberosities [13,18,19,20] or pubococcygeal line (PCL) [2,3,4,6,12,21,22,23,24,25]. Several studies measure perineal descent as the difference in position of the ARJ, rather than the difference compared to a reference line, however a wide range of cut-off values [2 to 4 cm] are reported [8,13,14,15,26,27,28,29,30,31,32]. Experts conclude that dynamic descent is best quantified by assessing the movement of the ARJ by measuring the difference between the ARJ position at rest and the point of maximum descent during evacuation [16]. The poor consensus in the literature on how to measure perineal descent and which cut-off to use calls for standardisation [27].

To allow standardisation and comparison between imaging techniques, the definition of perineal descent should be applicable for all techniques. It is not possible to visualise the PCL on ultrasound, therefore the level of the ARJ at rest and at maximum Valsalva cannot be measured. However, assessing the movement of the ARJ could potentially be performed on ultrasound as it does not require a point of reference. Pelvic floor ultrasound has recently been validated for diagnosing posterior pelvic floor disorders in women with ODS [33,34], however measuring perineal descent remains to be established for the use with pelvic floor ultrasound, including defining cut-off values. Moreover, the diagnostic test accuracy (DTA) of all imaging methods needs to be established as a recent meta-analysis was not able to draw conclusions regarding the DTA of imaging for perineal descent due to lack of data [33].

The aim of this study is to validate a uniform method for measuring perineal descent which can be used for all imaging techniques, to establish cut-off values for this measurement and to assess DTA using these cut-off values. Secondly, to correlate perineal descent to symptoms, signs and imaging findings in women with ODS to assess its clinical relevance.

## 2. Methods

### 2.1. Patient Recruitment

Consecutive women with any symptoms of ODS were prospectively recruited between January 2014 and January 2015 from colorectal surgery and urogynaecology clinics at Croydon University Hospital, United Kingdom. The current cross-sectional study is part of a cohort study aiming to assess the DTA of magnetic resonance imaging (MRI), evacuation proctography (EP) and pelvic floor ultrasound for diagnosis of posterior pelvic floor disorders [34]. Exclusion criteria were age younger than 18 years, not being able to understand English, and lacking mental capacity to understand the study. Women with previous pelvic floor surgery were not excluded. 

### 2.2. Data Collection

Demographics were recorded including age, ethnicity, parity, body mass index, and previous pelvic floor surgery. Validated questionnaires were used to evaluate the women’s symptoms. For symptoms of ODS, the Renzi questionnaire was used. The Renzi score consists of five questions with answers ranging from never (0) to always (4), and total score ranging from 0 to 20 [35]. St Mark’s incontinence scoring system was used to assess faecal incontinence, with a total score range of 0–24 [36]. The pelvic organ prolapse symptom score (POPS-S) was used to assess symptoms of pelvic organ prolapse (POP); with a total score range of 0–28 [37]. POP on clinical examination was assessed using the validated International Continence Society POP quantification (ICS POP-Q) system [38]. The time interval between the four imaging techniques was kept as short as possible with a maximum of three months and with the restriction that no significant events that could alternate the pelvic floor should have happened in between (e.g., pelvic floor surgery, childbirth).

### 2.3. Imaging Acquisition

EP was performed by an experienced radiologist (A.St.) with the patient in the upright position sitting on a radiolucent commode. The small bowel and rectum were opacified with oral and rectal barium contrast. MRI was performed by specifically trained radiographers using a closed 1.5T magnet MRI scanner (Siemens Avanto; Siemens Healthcare, Erlangen, Germany). The rectum was filled with ultrasound gel. T2-weighted fast acquisition (TrueFISP) images were obtained with the patient in the supine position with the knees and hips flexed to facilitate evacuation of contrast. EP and MRI images were recorded in the sagittal plane at rest, squeeze, maximum Valsalva, and evacuation of the contrast. Pelvic floor ultrasound was performed by an experienced ultrasonographer (I.v.G) using a ProFocus ultrasound machine (BK Medical, Herlev, Denmark). The patient was in the supine position with the legs semiflexed and no contrast was used. Transperineal ultrasound (TPUS) was performed with a curved-array transducer (Type 8802, 3.5–6.0 MHz, focal range 10–135 mm) placed on the perineum in a vertical position showing in the midsagittal plane the bladder, vaginal walls and rectum. During Valsalva the probe gently followed the movement of the perineum. Endovaginal ultrasound (EVUS) was performed with a high-resolution linear transducer (Type 8838, 12–6 MHz, focal range 3–60 mm, contact surface 65 mm) placed into the vagina facing the posterior vaginal wall visualising the posterior pelvic floor in the midsagittal plane. The probe was kept as steady as possible into the vagina during the patient’s Valsalva manoeuvre. For both TPUS and EVUS, images were acquired at rest, squeeze, and maximum Valsalva and stored as cineloops. At least three cineloops were stored to ensure the patient performed maximum Valsalva.

### 2.4. Imaging Analysis

**Figure 1:**
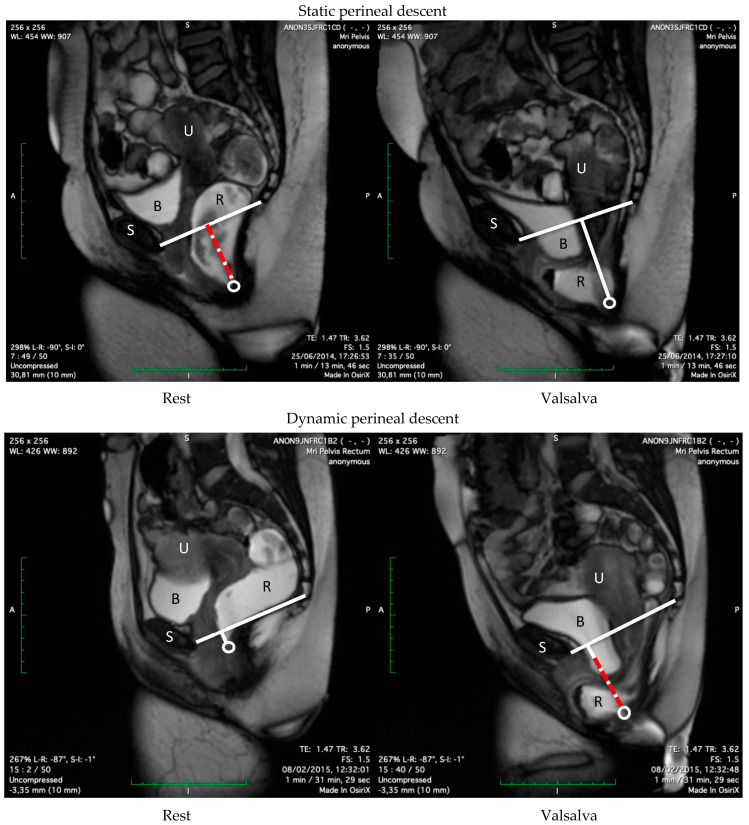
Perineal descent on MRI: *Static* perineal descent (red dashed) i.e., large difference between position of the ARJ and the PCL at rest, with subsequent minimal difference in movement of the ARJ during maximum Valsalva. *Dynamic* perineal descent (red dashed) i.e., large difference in movement of the ARJ during maximum Valsalva, following a minimal difference between position of the ARJ and the PCL at rest. S = symphysis pubis, B = bladder, R = rectum, U = uterus, circle = ARJ.

**Figure 2:**
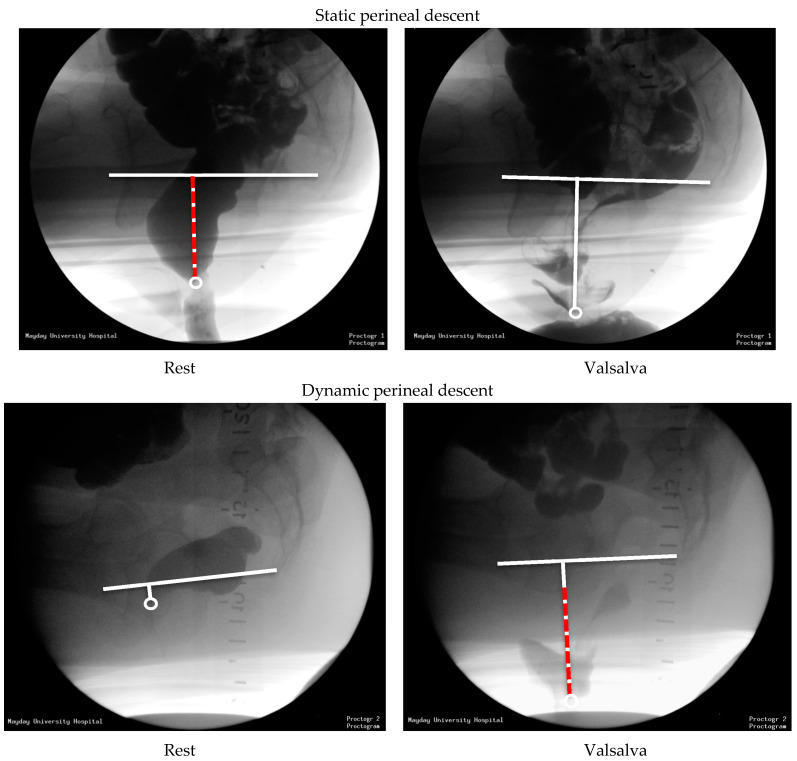
Perineal descent on EP: *Static* perineal descent (red) i.e., large difference between position of the ARJ (circle) and the PCL at rest, with subsequent minimal difference in movement of the ARJ (circle) during maximum Valsalva. *Dynamic* perineal descent (red) i.e., large difference in movement of the ARJ during maximum Valsalva, following a minimal difference between position of the ARJ and the PCL at rest.

**Figure 3:**
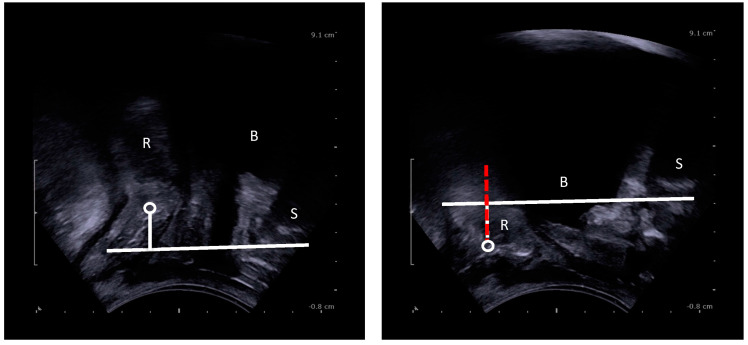
Dynamic perineal descent on TPUS (red dashed): i.e., difference in movement of the ARJ between rest and maximum Valsalva. S = symphysis pubis, B = bladder, R = rectum, circle = ARJ.

**Figure 4:**
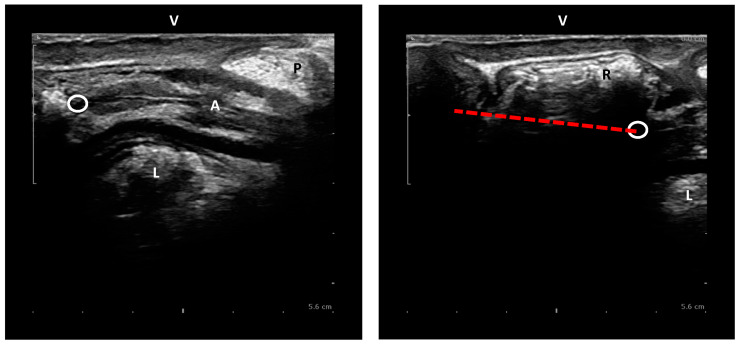
Dynamic perineal descent on EVUS (red dashed): i.e., difference in movement of the ARJ between rest and maximum Valsalva. P = perineal body, L = Levator ani muscle, A = Anal canal, R = Rectum, V = Vagina, circle = ARJ.

The anorectal junction (ARJ) is defined as the point of intersection between the central axis of the anal canal and the line along the posterior wall of the distal rectum [12,19]. The pubococcygeal line (PCL) is a straight line drawn from the inferior border of the symphysis pubis to the lowest (horizontal) joint of the os coccyx [11,22]; negative numbers are results above and positive numbers are results below the PCL. Perineal descent is assessed on EP and MRI in the midsagittal plane by measuring the position of the ARJ perpendicular from the PCL at rest (i.e., *static* descent) and during evacuation (i.e., descent *at Valsalva*) [3,4,6,23,25]. The difference between the ARJ position at rest and the point of maximum descent was calculated (i.e., *dynamic* descent) [15,28,31,39] (Figure 1 and Figure 2). On pelvic floor ultrasound (EVUS and TPUS), dynamic descent was assessed in the midsagittal plane playing the 2D cineloops of the collected ultrasound data. To measure dynamic descent on TPUS, the position of the ARJ compared to the symphysis pubis at rest and at maximum Valsalva was calculated (Figure 3). To measure dynamic descent on EVUS, the location of the ARJ at rest was marked and subsequently the cineloop was started and stopped again at the point of maximum Valsalva where the ARJ was marked to calculate the distance of the ARJ between rest and Valsalva (Figure 4). Offline analysis of EP and MRI images was performed by I.v.G and A.St using Sectra PACS software (Linköping, Sweden). TPUS and EVUS were analysed by I.v.G. and K.K using VirtualDub software (version 1.10.4) with the aid of Meazure© software (version 2.0.1). All observers were blinded to clinical findings and results of the other imaging techniques.

For the correlation between perineal descent and findings on imaging in women with ODS, the presence and absence of the other posterior pelvic floor disorders were assessed by two blinded observers using pre-defined cut-off values and discrepancies were solved by a third observer as described in the parent study [34]. Rectocele was present when its depth was >20 mm measured perpendicular from the expected contour of the anterior rectal wall on EP and MRI and >10 mm on TPUS and EVUS. An enterocele was present on EP and MRI if small bowel was visualised below the PCL and on TPUS and EVUS if small bowel was visualised near the rectovaginal septum. Intussusception was present on all four imaging techniques when a full-thickness circumferential invagination was visualised at Valsalva. Anismus was present when a paradoxical contraction of the puborectalis muscle was visualized during Valsalva. The true patient status for presence of posterior pelvic floor disorders was defined by combining the results of all four diagnostic techniques through a statistical model (latent class analysis), because the outcome of the different imaging techniques may vary. Major Levator Ani Muscle (LAM) avulsion was assessed on MRI and defined as either unilateral complete avulsion or bilateral avulsion (≥50% of the muscle bulk missing bilateral) [40]. Hiatal area was measured in the axial plane on MRI in the plane of the minimal hiatal dimensions at rest.

### 2.5. Statistical Analysis

Interobserver agreement for the different perineal descent measurements was assessed with the intraclass correlation coefficient (ICC) from a two-way random-effects model and a consistency definition (<0.50 poor; 0.50–0.75 moderate, 0.75–0.90 good and >0.90 excellent repeatability) [41]. The final measurement for each patient and on each imaging technique was calculated as the average of the two observers. Agreement between imaging techniques for the different perineal descent measurements was assessed using Bland–Altman limits of agreement [42]. The mean difference (δ) between the measurements of two different imaging techniques represents the systematic bias between those imaging techniques. The standard deviation of the differences (SDd) was used to construct limits of agreement (LOAs = δ ± 1.96 × SDd).

To establish cut-off values, EP was used as a reference standard since this imaging technique is validated and has most experience in measuring perineal descent. To define a cut-off value for dynamic descent on EP, Receiver Operating Characteristics (ROC) curves were created using cut-off values from literature for presence of descent at Valsalva on EP. By maximising the Youden’s index, e.g., the point of maximum combined sensitivity and specificity, a cut-off value for dynamic descent on EP was established. For each of the imaging techniques, we evaluated the relationship between measurements, using EP as the independent variable and the other imaging technique as the dependent variable. Since all relationships appeared to be linear, we used linear regression to quantify them and we estimated the cut-offs for the other imaging techniques by predicting which values corresponded best to the cut-off of EP.

DTA of imaging techniques for dynamic descent using the new cut-off values was assessed using latent class analysis (LCA) in the absence of a reference standard [43]. In LCA, the results of the imaging techniques are assumed to be imperfect observations of the true unobserved status of the patients, i.e., latent classes ‘healthy’ and ‘diseased’. Sensitivity, specificity, area under the ROC curve (AUC), positive predictive value (PPV), negative predictive value (NPV), likelihood ratio positive (LR+) and likelihood ratio negative (LR-) values with 95% Credibility Intervals (CrI’s) were estimated using a Bayesian approach to LCA with non-informative priors for sensitivity and 1-specificity for all imaging techniques and a prior prevalence of 50%, all based on beta-distributions B(1,1). We assumed conditional independence, because imaging analysis of the four imaging techniques was performed blinded. Results were calculated with the R package BayesLCA (version 19) [44], using Gibbs sampling to sample from the parameters’ true distribution. We used Markov Chain Monte Carlo (MCMC) with a burn-in of 5000 samples, 100,000 iterations and thinning of 20. Model fit was checked by the raftery.diag statement from the coda package (version 0.19-4) [45], and by evaluating the following plots (from package BayesLCA): a plot summarising item and class probability, a mosaic plot representing classification uncertainty, a plot with item probability density estimates, a plot with conditional class probability density estimates, and a diagnostics plot.

The Spearman correlation coefficient was used to explore the correlation of perineal descent measurements on the different imaging methods to symptoms and signs of obstructed defaecation syndrome. With the independent *t*-test, the association between perineal descent measurements and presence/absence of rectocele, enterocele, intussusception, anismus and major LAM avulsion was assessed. A two-sided *p*-value < 0.05 was considered statistically significant.

The sample size calculation was performed for the parent study based on the expected prevalence, sensitivity and specificity [34]. Statistical analyses were performed using SPSS Statistics version 26 (IBM Corp., Armonk, NY, USA), and the statistical package R, version 4.2.0 [46].

## 3. Results

### 3.1. Baseline Characteristics

A total of 131 consecutive women with symptoms of ODS were recruited in the parent study [34]. Mean age was 54 (SD 14, range 25–90) years, mean body mass index was 27 (SD 5, range 17–47) kg/m^2^ and mean parity was 2.3 (SD 1.3, range 0–6). Ethnicity was 77% Caucasian, 15% Afro-Caribbean and 8% Asian. Previous POP surgery had been performed in 24 (18%) women, 39 (30%) had a hysterectomy, and 12 (9%) had previous surgery for obstructed defaecation syndrome (six stapled transanal rectal resections, four rectopexies and two had both). All women underwent EP and pelvic floor ultrasound. Nine women did not have MRI because of contra-indications or were not willing to undergo this examination. The median time difference between EP and MRI was 11.5 days (range 0–92 days), between EP and ultrasound 3.0 days (range 0–58 days), and between MRI and ultrasound 8.5 days (range 0–89 days). For EP a cineloop of one patient and for TPUS cineloops of four patients were not analysable. All MRI and EVUS cineloops were analysable.

### 3.2. Agreement Between Observers and Imaging Methods

Interobserver agreement of the perineal descent measurements is presented in Table 1. For measuring dynamic perineal descent, MRI and EVUS show a good interobserver agreement (ICC 0.86 and 0.85, respectively) and EP and TPUS a moderate interobserver agreement (ICC 0.61 and 0.59, respectively). Interobserver agreement of dynamic descent was similar to static descent and descent at maximum Valsalva on EP (ICC 0.57–0.62) and MRI (ICC 0.85–0.92). Table 2 presents differences in measurements between imaging techniques using Bland-Altman LOA and ICC. For dynamic descent, agreement was moderate between EP and MRI (ICC 0.69) and between TPUS and EVUS (ICC 0.73). Agreement between MRI and ultrasound was not significantly higher (ICC 0.54–0.56) than between EP and ultrasound (ICC 0.37–0.46). The standard deviations of differences for nearly all measurements were higher than 10 mm, leading to wide LOAs and moderate ICCs. Systematic bias (δ) between EP and MRI was 15 mm, between MRI and ultrasound approximately 20 mm and between EP and ultrasound approximately 5 mm. Systematic bias between the two ultrasound methods was only 0.6 mm.

### 3.3. Cut-Off Values

ROC curves for the most used cut-off values in literature for descent at Valsalva, i.e., ARJ > 30 mm, >40 mm and >50 mm below the PCL line on EP, compared to dynamic descent on EP showed that a cut-off of 20 mm would provide the highest combination of sensitivity and specificity (Supplementary content Table S1) (Supplementary content Figure S1). Linear regression based on the 20 mm cut-off for EP resulted in a cut-off value for dynamic descent for MRI of 35 mm and for TPUS and EVUS of 15 mm. This cut-off value means that values below correspond to normal descent and above to abnormal perineal descent. The established cut-off values are similar to the systematic biases between imaging techniques as shown in Table 2.

### 3.4. Diagnostic Test Accuracy

Diagnostic test characteristics of dynamic descent for all imaging techniques using our established cut-off values and based on LCA are presented in Table 3. Prevalence of dynamic descent was 55% (CrI 24–77%). Sensitivity was moderate for EP (0.78; CrI 0.63–0.95) and MRI (0.74; CrI 0.57–0.95), fair for TPUS (0.65; CrI 0.50–0.89) and poor for EVUS (0.58; CrI 0.42–0.87). Specificity was similar for all imaging techniques (0.73–0.77). PPV’s and NPV’s were moderate for all imaging techniques.

### 3.5. Correlation to Clinical Findings

Age showed a positive correlation with static perineal descent on EP and MRI (*r* = 0.48 and *r* = 0.53, respectively), whereas age showed a negative correlation with dynamic descent on EP, MRI and TPUS (*r* = −0.41, −0.40 and −0.21, respectively), i.e., older women had more static descent and younger women had more dynamic descent (Table 4). BMI showed a positive correlation with static descent on EP and MRI (*r* = 0.18 and *r* = 0.22 respectively), but not for dynamic perineal descent, i.e., higher weight on the pelvic floor impacts the position of the perineum at rest. Parity showed a positive correlation to static descent on MRI (*r* = 0.25), however this correlation was not present for the other perineal descent measurements or imaging techniques.

Women with static descent on EP and MRI had fewer symptoms of ODS (*r* = −0.19 and −0.20), and women with dynamic descent on EP and MRI had more symptoms of ODS (both *r* = 0.21) (Table 4). Table 5 shows results of the individual questions. Women with dynamic descent on EP and MRI were more often straining excessively (*r* = 0.23 and 0.24) and used laxatives more often (*r* = 0.24). The most classic symptoms of ODS, being incomplete evacuation and the need to digitate, did not show any correlation to perineal descent measurements. Moreover, both ultrasound techniques did not show any correlation to symptoms of ODS.

Static descent on MRI correlated with symptoms of faecal incontinence and POP (*r* = 0.29 and 0.19), and POP in all three compartments (*r* = 0.19–36), whereas static descent on EP correlated to anterior and middle compartment prolapse (*r* = 0.25 and 0.28). Posterior vaginal wall prolapse correlated with dynamic descent on TPUS and EVUS (*r* = 0.32 and 0.26), however did not correlate to dynamic descent on EP and MRI.

### 3.6. Association to Imaging Findings

The association of perineal descent measurements with presence of other posterior compartment disorders is presented in Table 6. Both static and dynamic descent showed associations with presence of rectocele, enterocele and intussusception. Women with anismus had less static and dynamic descent than women without anismus. More women were able to evacuate >50% of the contrast with EP (65%) than with MRI (41%). Less perineal descent was found on MRI in women that were not able to expel more than half of the contrast, which was not found for EP. Women with a major LAM avulsion had more static descent on MRI, and LAM hiatal area correlated with static descent on EP and MRI; this correlation was not found for dynamic descent.

## 4. Discussion

To allow standardisation of perineal descent measurements, we validated a uniform method that can be applied to all available imaging techniques. Dynamic perineal descent could reliably be assessed by measuring the difference between the position of the ARJ at rest and Valsalva. The systematic differences in measurements between imaging techniques show the need for individual cut-off values. New established cut-off values for dynamic descent are for EP 20 mm, MRI 35 mm, TPUS 15 mm and EVUS 15 mm. Sensitivity was moderate for EP and MRI, fair for TPUS and poor for EVUS. Specificity was similar for all imaging techniques. Women with static perineal descent had more symptoms and signs of prolapse, and more often a LAM avulsion and larger hiatal area. Women with dynamic perineal descent reported more excessive straining and more often use of laxatives. Classic symptoms of ODS (incomplete evacuation and digitation) did not correlate to perineal descent measurements. Both static and dynamic perineal descent were associated with presence of rectocele, enterocele, intussusception, and absence of anismus.

A high interobserver agreement was found for MRI, in agreement with Broekhuis et al. [47]. However, agreement was moderate for EP. This might be a result of poorer visibility of bony structures on EP conversely to MRI, which causes difficulty in defining the PCL resulting in large differences in measurements between observers. Moreover, a systematic difference was found between EP and MRI of 15 mm, which could be explained by MRI performed in the supine and EP in the upright position. In the latter, the ARJ at rest is lower due to gravity, resulting in a smaller difference between rest and Valsalva measurements [48]. Interobserver agreement was higher for EVUS than for TPUS, but there was a good agreement between both ultrasound methods for measuring dynamic descent, with no systematic difference, showing they can be used interchangeably.

A recent Cochrane review, also using LCA to account for the imperfect reference standard, showed a sensitivity of 98% and specificity 83% for EP, and a sensitivity of 94% and specificity of 79% for MRI to diagnose perineal descent [33]. These figures are all higher than in this study, however most of the articles included in the review used a different definition of perineal descent, e.g., PCL with a range of cut-off vales 2, 2.5, 3 or 5 cm [6,7,12,14,24,25]. The cut-off value of dynamic descent established for EP in this study (20 mm) is the same as recently agreed in a consensus meeting of experts [16]. Moreover, dynamic descent of less than 20 mm is often seen in healthy volunteers using EP [49].

Similar to other studies [11,15,50,51], younger women showed more frequently an excessive movement of the rectum during evacuation, whereas older women had more often a low position of the rectum at rest with little movement during Valsalva. Women with dynamic descent had symptoms of functional constipation, whereas women with static descent reported more symptoms of POP and faecal incontinence, which is in agreement with others [11,50]. In addition, we found more often prolapse in all three pelvic floor compartments, and presence of LAM avulsion and enlarged hiatal area. The above findings strengthen the hypothesis of Bartolo et al. that perineal descent starts with dynamic descent due to excessive straining or obstetric trauma to the pelvic floor muscles, which, after decades, results in static descent with consequent POP and stretch injury to the pudendal nerves leading to faecal incontinence [4,5].

Typical symptoms of ODS did not correlate to perineal descent, whereas perineal descent was associated with the presence of rectocele, enterocele and intussusception, or absence of anismus, which is in agreement with findings of others [11,27,31,50,52,53,54]. This suggests that symptoms of ODS are probably caused by presence of the other posterior compartment disorders and not by pelvic floor descent in specific.

Perineal descent measured as the position of the ARJ below the PCL at maximum Valsalva does not truly reflect what happens to the ARJ *during* the Valsalva manoeuvre; if extensive perineal descent is present at rest, the ARJ consequently does not move during Valsalva, this would rather be *static* perineal descent than *dynamic* perineal descent [17,27]. We have shown that measuring the movement of the ARJ during Valsalva correlates similar or better to symptoms and signs of pelvic floor dysfunction than measuring ARJ position in relation to PCL at maximum Valsalva.

### 4.1. Implications for Clinical Practice

Dynamic descent could reliably be assessed by measuring the difference between the position of the ARJ at rest and Valsalva. We recommend the use of this uniform measurement to allow standardisation of all imaging techniques. Ideally, both static and dynamic descent should be analysed, because they have a different role in the pathophysiology of PFD. Timely identification is important to prevent excessive perineal descent and subsequent complete pelvic floor decompensation by early implementation of lifestyle changes (e.g., bowel rehabilitation, pelvic floor muscle exercises). In previous studies [33,34] we have shown that pelvic floor ultrasound could be used for the initial assessment of women presenting with symptoms of ODS, to assess presence of rectocele, enterocele, intussusception and animus. Pelvic floor ultrasound could be used as a triage test because of similar specificity to EP and MRI. Now we have shown that the specificity of dynamic pelvic floor descent for ultrasound was similar to EP and MRI, dynamic perineal descent could be added to the clinical implementation of pelvic floor ultrasound. Given that ultrasound is more accessible and less invasive compared to EP and MRI, future research should focus on how to improve its DTA to increase clinical utility. Moreover, the proposed cut-off values should be externally validated. Until then, perineal descent should be reported as a measurement value rather than as presence or absence using a cut-off value.

### 4.2. Strengths and Limitations

This is the first study to define how to measure perineal descent on ultrasound and to assess its DTA after establishing cut-off values. Measurements were performed by two blinded observers to reduce bias. The women included had mild to severe ODS symptoms, this range allows correlation of perineal descent severity to severity of ODS and PFD to define clinically relevant findings. In the absence of a reference standard for diagnosis of perineal descent, LCA has been used to assess DTA combining the results in a statistical model.

Ideally, DTA is assessed using pre-defined cut-off values. Assessing DTA after establishing cut-off values, could artificially increase sensitivity and specificity. Incomplete evacuation could influence the extent of perineal descent measurements. Women with more severe ODS might not be able to evacuate, resulting in less perineal descent, although supine position and anismus could also affect ability to evacuate. The position of the transperineal and endovaginal probe could restrict the extent of perineal descent, although care was taken to avoid this.

## 5. Conclusions

Dynamic perineal descent is a reliable measurement that can be applied to all imaging techniques to allow standardisation. The systematic differences, however, show that each imaging technique requires its own cut-off value. Static perineal descent is more often present in women with POP and dynamic perineal descent is more often present in women with constipation. Perineal descent does not correlate with typical symptoms of ODS. Specificity of TPUS and EVUS is comparable to EP and MRI, hence ultrasound could be used for the initial assessment of PFD.

## Figures and Tables

**Table 1:** Interobserver agreement of perineal descent measurements on all four imaging techniques.

Perineal Descent	EP		MRI		TPUS		EVUS	
	ICC (95% CI)	Mean Difference in mm (SD)	ICC (95% CI)	Mean Difference in mm (SD)	ICC (95% CI)	Mean Difference in mm (SD)	ICC (95% CI)	Mean Difference in mm (SD)
Static	0.57 (0.39–0.70)	8.2 (12.9)	0.85 (0.78–0.89)	−4.2 (9.2)	N/A	N/A	N/A	N/A
Valsalva	0.62 (0.46–0.74)	6.6 (13.3)	0.92 (0.89–0.95)	−2.8 (9.2)	N/A	N/A	N/A	N/A
Dynamic	0.61 (0.45–0.73)	−1.2 (13.1)	0.86 (0.81 −0.91)	1.2 (11.9)	0.59 (0.42–0.71)	3.7 (12.4)	0.85 (0.79–0.89)	3.1 (7.5)

Mean difference calculated as measurement observer 1 minus measurement observer 2. ICC, intra-class correlation coefficient (Two-way random effects model, consistency); CI, confidence interval; SD, standard deviation; N/A, not analysed.

**Table 2:** Agreement of perineal descent measurements between imaging techniques.

Imaging Techniques	Perineal Descent	Imaging A Mean (SD)	Imaging B Mean (SD)	Mean Difference (δ) (95% CI)	SDd	Lower LOA (95% CI)	Upper LOA (95% CI)	ICC (95% CI)
EP (A) vs. MRI (B)	Static	26.0 (10.3)	16.5 (11.7)	9.1 (8.1 to 10.1)	11.0	−12.5 (−13.5 to −11.5)	30.6 (29.7 to 31.6)	0.67 (0.53–0.77)
	Valsalva	47.2 (12.6)	53.1 (16.5)	−5.9 (−7.2 to −4.6)	14.2	−33.7 (−35.0 to −32.4)	21.9 (20.6 to 23.2)	0.70 (0.58–0.79)
	Dynamic	21.2 (10.8)	36.6 (16.4)	−15.1 (−16.3 to −13.8)	13.6	−41.8 (−43.0 to −40.5)	11.7 (10.4 to 12.9)	0.69 (0.55–0.78)
EP (A) vs. TPUS (B)	Dynamic	21.2 (10.8)	15.3 (9.7)	5.8 (4.7 to 6.9)	12.1	−18.0 (−19.1 to −16.9)	29.6 (28.5 to 30.7)	0.46 (0.24–0.62)
EP (A) vs. EVUS (B)	Dynamic	21.2 (10.8)	14.9 (11.3)	6.3 (5.1 to 7.5)	13.9	−20.9 (−22.1 to −19.6)	33.4 (32.2 to 34.7)	0.37 (0.10–0.55)
MRI (A) vs. TPUS (B)	Dynamic	36.6 (16.4)	15.3 (9.7)	20.7 (19.1 to 22.1)	15.1	−8.9 (−10.3 to −7.5)	50.4 (49.0 to 51.8)	0.54 (0.34–0.68)
MRI (A) vs. EVUS (B)	Dynamic	36.6 (16.4)	14.9 (11.3)	21.5 (20.0 to 22.9)	15.7	−9.3 (−10.7 to −7.8)	52.2 (50.8 to 53.6)	0.56 (0.38–0.70)
TPUS (A) vs. EVUS (B)	Dynamic	15.3 (9.7)	14.9 (11.3)	0.6 (−0.3 to 1.4)	9.6	−18.2 (−19.1 to −17.4)	19.4 (18.6 to 20.3)	0.73 (0.62–0.81)

Difference between measurements is calculated as measurement A minus measurement B; δ, mean difference between measurements; SDd, standard deviation of difference between measurements; LOA, limits of agreement (calculated as mean difference (δ) ± 1.96 × SDd); SD, standard deviation; ICC, intra-class correlation coefficient (Two way mixed model, consistency); CI, confidence interval.

**Table 3:** Diagnostic test accuracy of *dynamic* perineal descent of all four imaging techniques using the new cut-off values.

Imaging Technique	Cut-Off Value	Sensitivity (95% CrI)	Specificity (95% CrI)	AUC (95% CrI)	PPV (95% CrI)	NPV (95% CrI)	LR+ (95% CrI)	LR− (95% CrI)
EP	20 mm	78.4 (63.6–94.5)	73.0 (50.7–97.0)	76.1 (61.9–89.2)	78.4 (36.9–98.4)	74.3 (45.6–95.8)	1.52 (0.93–16.34)	0.16 (0.02–0.52)
MRI	35 mm	74.3 (57.8–95.3)	76.2 (56.3–96.7)	75.8 (62.3–88.0)	79.5 (41.3 −98.2)	71.1 (42.0–97.1)	1.71 (0.95–16.90)	0.19 (0.02 –0.66)
TPUS	15 mm	65.3 (49.8–89.5)	76.2 (58.7–95.9)	71.5 (58.6–84.0)	77.0 (42.4–97.2)	64.5 (36.1–94.1)	1.77 (0.94–13.32)	0.31 (0.05–0.96)
EVUS	15 mm	58.2 (41.7–87.1)	77.5 (61.7–93.3)	68.5 (55.5–82.9)	75.4 (45.9–94.7)	60.4 (31.8–94.2)	1.83 (0.96–8.58)	0.40 (0.05–1.35)

Dynamic perineal descent: difference in position of the ARJ between rest and Valsalva. Cut-off value: movement of ARJ in mm above which presence of dynamic perineal descent is defined. Results based on Latent Class Analysis. CrI, Credibility interval; AUC, area under the curve; PPV, positive predictive value; NPV, negative predictive value; LR+, positive likelihood ratio; LR−, likelihood ratio.

**Table 4:** Correlation between perineal descent measurements and clinical findings.

Variable	Range	Mean (SD)	EP			MRI			TPUS	EVUS
			Static	Valsalva	Dynamic	Static	Valsalva	Dynamic	Dynamic	Dynamic
Age	25 to 90	53.6 (14.3)	0.48 **	0.06	−0.41 **	0.53 **	−0.02	−0.40 **	−0.21 *	−0.11
BMI	17 to 47	26.7 (4.9)	0.18 **	0.23 **	0.04	0.22 *	0.30 **	0.09	−0.00	0.03
Parity	0 to 6	2.3 (1.3)	0.14	0.03	−0.13	0.25 **	0.14	−0.05	−0.03	0.00
Renzi questionnaire total	0 to 18	8.84 (4.3)	−0.19 *	0.00	0.21 *	−0.20 *	0.10	0.21 *	0.09	0.11
St Marks score total	0 to 24	5.53 (6.1)	0.03	−0.07	−0.16	0.29 **	−0.00	−0.22 *	−0.10	−0.09
POPSS total	0 to 28	8.54 (5.5)	−0.08	−0.03	0.03	0.19 *	0.10	−0.06	0.02	0.08
POP-Q Ba (cm)	−3 to 3	−2.1 (1.1)	0.25 *	0.16	0.01	0.19 *	0.17	0.06	0.07	0.25 **
POP-Q C (cm)	−10 to 10	−6.1 (2.4)	0.28 **	0.09	−0.15	0.36 **	0.16	−0.08	0.05	0.27 **
POP-Q Bp (cm)	−3 to 3	−2.1 (1.3)	0.04	0.14	0.05	0.21 *	0.28 **	0.11	0.32 **	0.26 **
LAM Area (cm^2^)	7 to 27	14.7 (4.0)	0.30 **	0.16	−0.11	0.51 **	0.19 *	−0.19 *	−0.05	0.01

Perineal descent: Static, ARJ below PCL at rest; Valsalva, ARJ below PCL at Valsalva; Dynamic, difference between ARJ at rest and Valsalva. Results are stated as Spearman Correlation Coefficients. * = correlation is significant at the level of 0.05 (2-tailed). ** = correlation is significant at the level of 0.01 (2-tailed).

**Table 5:** Correlation between perineal descent on all four imaging techniques and individual questions of ODS questionnaire.

ODS Questionnaire	Mean (SD)	EP			MRI			TPUS	EVUS
		Static	Valsalva	Dynamic	Static	Valsalva	Dynamic	Dynamic	Dynamic
Excessive straining	1.28 (1.4)	−0.14	0.04	0.23 *	−0.20 *	0.14	0.24 **	0.03	0.13
Incomplete rectal evacuation	3.01 (1.2)	−0.10	−0.15	−0.07	−0.09	−0.12	−0.04	0.04	0.05
Use of enema’s/laxatives	1.05 (1.6)	0.03	0.13	0.17	−0.11	0.13	0.24 **	0.08	0.11
Vaginal/perineal digital pressure	1.86 (1.7)	−0.12	0.03	0.16	−0.11	0.17	0.18	0.08	0.07
Abdominal discomfort/pain	1.63 (1.5)	−0.23 **	0.00	0.15	−0.10	−0.00	0.04	0.10	−0.02

Perineal descent: Static, ARJ below PCL at rest; Valsalva, ARJ below PCL at Valsalva; Dynamic, difference between ARJ at rest and Valsalva. Results are stated as Spearman Correlation Coefficients. Each question range from 0 to 4, total range 0 to 20. * = correlation is significant at the level of 0.05 (2-tailed), ** = correlation is significant at the level of 0.01 (2-tailed).

**Table 6:** Association between perineal descent measurements and presence of imaging findings in women with ODS.

Variable	Number	EP			MRI			TPUS	EVUS
		Static	Valsalva	Dynamic	Static	Valsalva	Dynamic	Dynamic	Dynamic
Rectocele ^	Present *n* = 54	26.5 (9.6)	51.2 (12.0) **	24.8 (10.7) **	19.0 (11.1) *	60.4 (11.7) **	41.3 (13.3) **	19.3 (9.9) **	19.0 (11.0) **
	Absent *n* = 68	25.0 (10.7)	44.0 (12.8)	19.1 (10.5)	14.6 (12.0)	47.4 (17.5)	32.8 (17.6)	13.0 (8.7)	12.1 (11.3)
Enterocele ^	Present *n* = 20	29.7 (5.5) *	53.9 (11.6) *	24.1 (11.4)	21.4 (8.8) *	63.0 (14.2) **	41.6 (17.1)	20.3 (13.7)	25.5 (18.0) **
	Absent *n* = 102	24.8 (10.8)	45.9 (12.8)	21.1 (10.9)	15.6 (12.0)	51.2 (16.3)	35.6 (16.1)	15.0 (8.8)	13.1 (8.8)
Intussusception ^	Present *n* = 43	28.0 (9.0)	51.4 (9.8) **	23.4 (10.8)	20.2 (10.5) *	61.2 (12.7) **	41.0 (15.2) *	19.6 (12.0) **	21.0 (14.3) **
	Absent *n* = 79	24.3 (10.6)	44.9 (13.8)	20.6 (11.0)	14.6 (11.9)	48.7 (16.7)	34.2 (16.6)	13.7 (7.6)	11.9 (8.4)
Anismus ^	Present *n* = 10	19.9 (14.4)	35.9 (16.4) **	16.1 (13.9)	7.3 (8.6) **	28.1 (20.0) **	20.9 (17.5) **	6.4 (4.4) **	3.7 (3.9) **
	Absent *n* = 112	26.2 (9.7)	48.2 (12.1)	22.1 (10.6)	17.4 (11.7)	55.4 (14.2)	38.0 (15.6)	16.6 (9.7)	16.1 (11.6)
Evacuation of contrast > 50%	Present *n* = 85/49	27.3 (10.0)	48.4 (11.9)	21.1 (10.2)	19.7 (10.9) **	60.0 (10.3) **	40.4 (13.4) *	N/A	N/A
	Absent *n* = 46/70	23.7 (10.4)	45.1 (13.7)	21.4 (12.1)	14.1 (11.9)	48.3 (18.5)	34.2 (18.0)		
Major LAM avulsion MRI	Present *n* = 19	30.0 (7.7)	48.6 (8.0)	18.5 (9.5)	23.4 (12.5) **	54.9 (9.4)	31.6 (11.6)	15.4 (8.6)	11.4 (7.4)
	Absent *n* = 104	25.0 (10.6)	47.0 (13.6)	22.0 (11.2)	15.3 (11.2)	52.8 (17.5)	37.5 (17.0)	15.8 (9.9)	15.8 (12.1)

Results are in millimetres expressed in mean with standard deviation. Perineal descent: Static, ARJ below PCL at rest; Valsalva, ARJ below PCL at Valsalva; Dynamic, difference between ARJ at rest and Valsalva. ^ = Presence or absence of rectocele, enterocele, intussusception and anismus is calculated from results of EP, MRI, TPUS and EVUS using LCA (latent class analysis). An independent samples *t*-test was used to calculate the difference between the average of perineal descent measurements (scale) between presence and absence of the condition (nominal). * = significant result at the level of 0.05 (2-tailed) ** = significant result at the level of 0.01 (2-tailed).

## Data Availability

The original contributions presented in this study are included in the article/Supplementary Material. Further inquiries can be directed to the corresponding author.

## Supplementary Materials

The following supporting information can be downloaded at: https://www.mdpi.com/article/10.3390/jcm14020548/s1, Figure S1: ROC curves for different cut-off values for perineal descent on EP at Valsalva. (A) >30 mm below PCL, (B) >40 mm below PCL, (C) >50 mm below PCL; Table S1: Highest Youden index based on ROC curves to establish new cut-off value for dynamic perineal descent on EP based on currently used cut-off values.
