# Supplementary material for: Evaluation of Perineal Descent Measurements on Pelvic Floor Imaging
Source: J Clin Med. 2025 Jan 16;14(2):548. doi: 10.3390/jcm14020548 (PMC11766426; doi:10.3390/jcm14020548)
Supplement: Supplementary file 1 [file jcm-14-00548-s001.zip › jcm-3377136-supplementary.pdf]

Journal of
Clinical Medicine

MDPI

Supplementary content Figure S1 ROC curves for different cut-off values for perineal descent on EP at Valsalva. (A) > 30 mm below PCL, (B) > 40 mm below PCL, (C) > 50 mm below PCL.

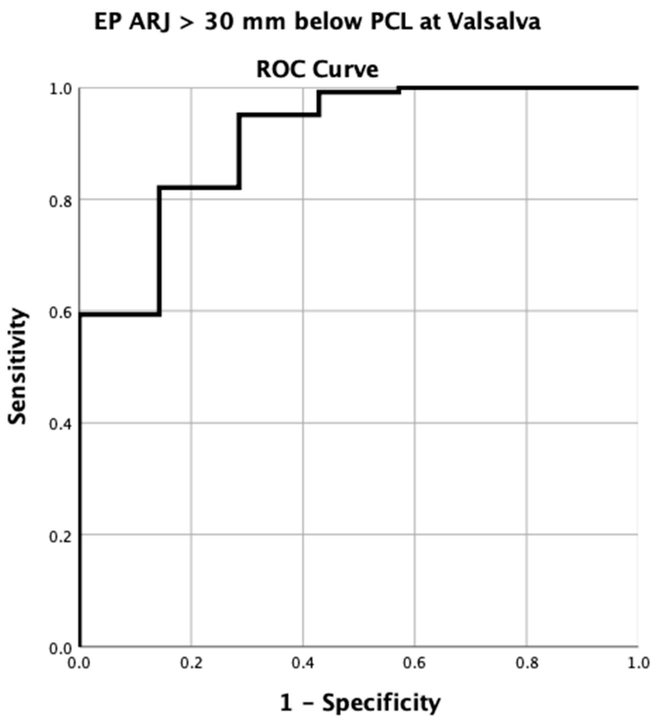

A

B

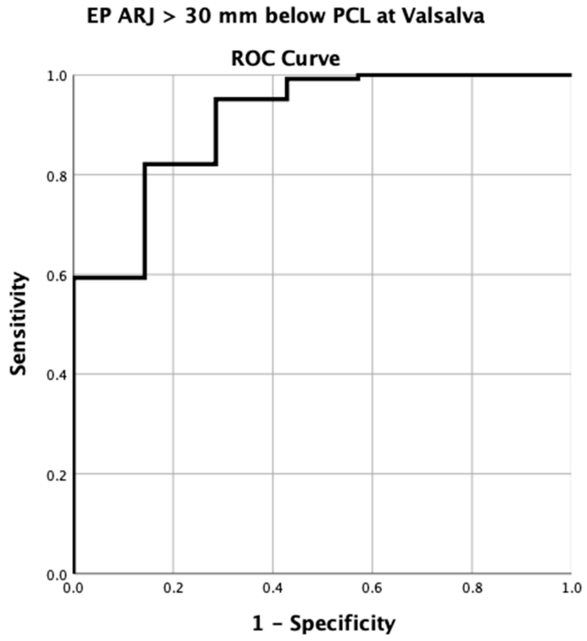

C

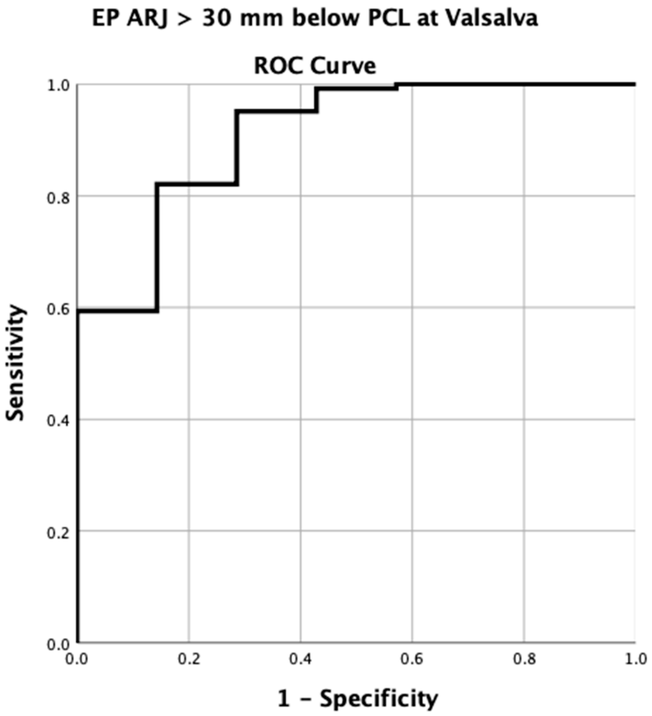

Supplementary content Table S1 Highest Youden index based on ROC curves to establish new cut-off value for *dynamic* perineal descent on EP based on currently used cut-off values.

Cut-off value	N pos	N neg	AUC	Sensitivity	Specificity	Youden index	Cut-off (mm)
**EP cut off > 30 mm below PCL**	123	7	0.908	0.593	1.000	0.593	19.65
				0.585	1.000	0.585	19.73
				0.577	1.000	0.577	19.98
**EP cut off > 40 mm below PCL**	107	23	0.765	0.626	0.826	0.452	19.98
				0.617	0.826	0.443	20.28
				0.607	0.826	0.433	20.35
**EP cut off > 50 mm below PCL**	54	76	0.718	0.722	0.632	0.354	20.58
				0.722	0.632	0.354	20.70
				0.704	0.658	0.362	20.73

ROC curves established using as nominal variable the current cut-off value and as test variable the measurement 'difference in perineal descent between rest and Valsalva' on evacuation proctography; EP, evacuation proctography; AUC, area under the curve; PCL, pubococcygeal line; Youden index calculated as sensitivity + specificity – 1
